# A Case Report of Pulmonary Alveolar Proteinosis Masquerading as Respiratory Distress Syndrome in Preterm Neonates

**DOI:** 10.7759/cureus.63866

**Published:** 2024-07-04

**Authors:** Aditi Rawat, Sagar Karotkar, Mahaveer S Lakra, Snehlata Hingway, Revatdhamma Meshram, Amar Taksande

**Affiliations:** 1 Department of Neonatalogy, Datta Meghe Institute of Higher Education and Research (Deemed to be University), Wardha, IND; 2 Department of Pediatrics, Datta Meghe Institute of Higher Education and Research (Deemed to be University), Wardha, IND; 3 Department of Pathology, Jawaharlal Nehru Medical College, Datta Meghe Institute of Higher Education and Research (Deemed to be University), Wardha, IND; 4 Department of Pediatrics, Jawaharlal Nehru Medical College, Datta Meghe Institute of Higher Education and Research (Deemed to be University), Wardha, IND

**Keywords:** rare genetic disease, respiratory distress in newborn, neonate, lung biopsy, pulmonary alveolar proteinosis

## Abstract

A rare and challenging case of a preterm neonate with clinical and radiological signs of respiratory distress syndrome (RDS) since the first hour of life but was refractory to its standard treatment regimes like surfactant therapy and ventilation. Postmortem lung biopsy led us to the diagnosis of congenital pulmonary alveolar proteinosis (PAP). It occurs due to the aggregation of abnormal surfactant proteins and lipids in the alveoli, which hampers gas diffusion across the alveoli. It presents as respiratory distress at birth, and its diagnosis is often missed due to its resemblance with RDS. Although the exact etiology remains elusive, mutations in genes encoding surfactant and granulocyte-macrophage colony-stimulating factor (GM-CSF) pathway components have been implicated in the pathogenesis of PAP. Treatment options are limited and only supportive. Among all these, whole-lung lavage is the most widely used management modality but with limited success in neonates.

## Introduction

Neonatal pulmonary alveolar proteinosis (PAP) is a rare and potentially life-threatening condition that affects newborns. It is distinguished by the collection of a periodic acid-Schiff (PAS)-positive diastase-resistant amorphous proteinaceous material in the alveoli leading to respiratory insufficiency [1]. Two types of PAP have been described; congenital PAP which has a fulminant course and is usually fatal, and other one is late-onset PAP which is less severe and mimics the adult form [2]. The clinical presentation at birth is nonspecific ranging from asymptomatic to progressive respiratory failure. The diagnosis is confirmed by lung biopsy and bronchoalveolar lavage [3]. Treatment options are limited with poor success rates including whole-lung lavage, lung transplantation, administration of granulocyte-macrophage colony-stimulating factor (GM-CSF), and gene therapy [4]. Here, we present a case of a severe form of congenital PAP presenting at birth as respiratory distress refractory to standard treatment.

## Case presentation

A 1.3 kg female child was born to a 28-year-old multigravida mother with a bad obstetric history at 28 weeks of gestation by cesarean section in view of fetal distress. Antenatal history revealed four previous neonatal deaths out of which one was stillbirth and the rest all succumbed within 24 hours of life. All previous pregnancies were preterm births, and the babies had respiratory distress at birth. The mother had a unicornuate uterus which was hypothesized to be the cause for premature deliveries. There was a history of third-degree consanguinity present. The antenatal period was uneventful till 28 weeks when the patient started experiencing preterm labor for which she was admitted, antenatal steroid coverage with intravenous (IV) dexamethasone was given, and owing to fetal compromise, she was taken up for emergency cesarean.

**Figure 1:**
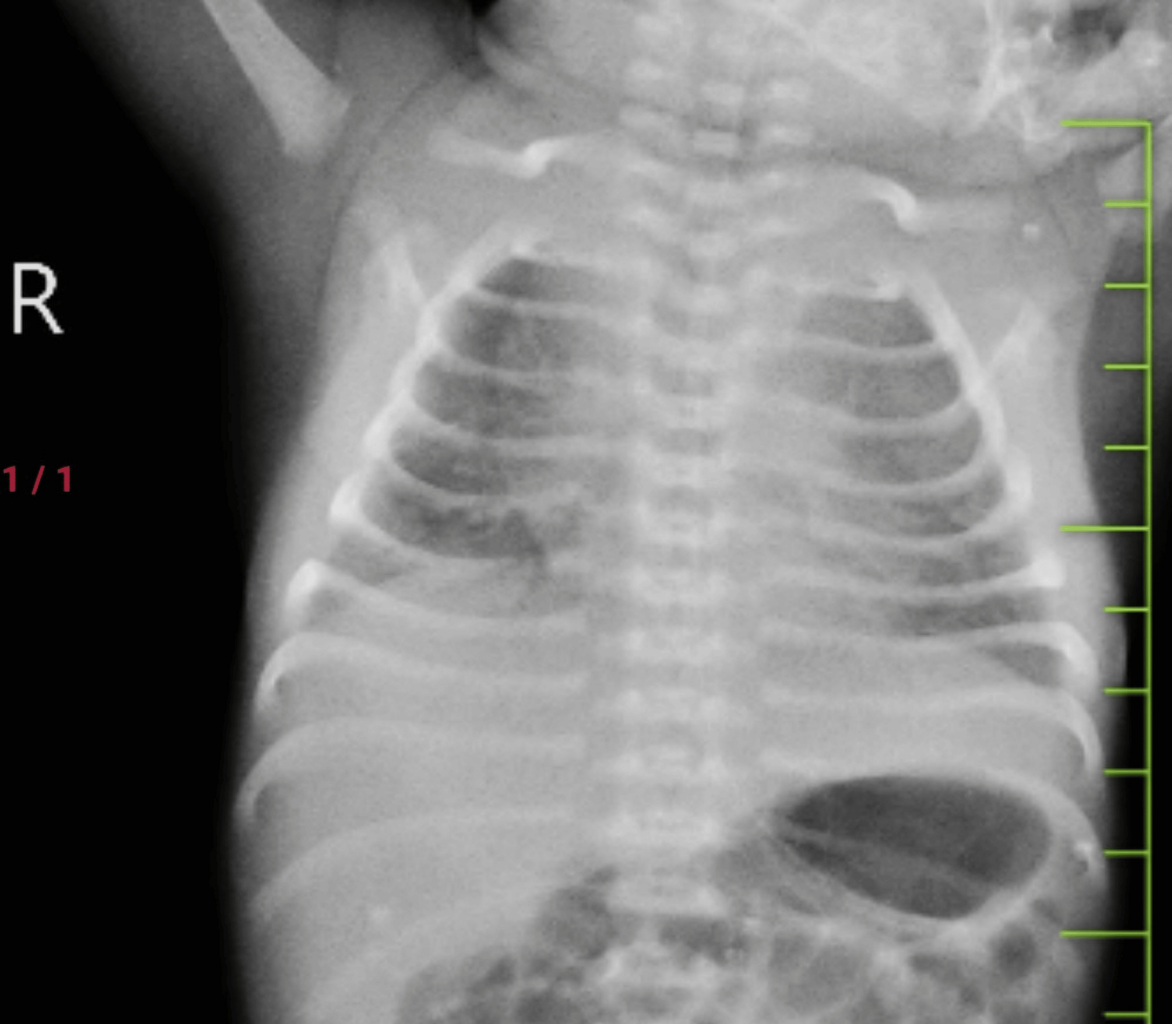
Low lung volume with bilateral homogenous ground glass opacities in lung fields suggestive of respiratory distress syndrome

The baby had severe respiratory distress at birth requiring delivery room continuous positive airway pressure (CPAP). In the neonatal intensive care unit (NICU), the baby was kept on CPAP support with a fraction of inspired oxygen (FiO2) requirement of 30% and a positive end-expiratory pressure (PEEP) of 5. Total parenteral nutrition and first-line antibiotics were initiated. The baby had a Silverman Anderson score of 5 with a chest x-ray suggestive of low lung volume and bilateral homogenous ground glass appearance suggestive of respiratory distress syndrome (RDS) as seen in Figure 1.

In view of the increasing Fio2 requirement of >30%, Neosurf was administered within two hours of life as standard treatment protocol for RDS. In view of persistent respiratory distress, the baby was intubated by the fourth hour of life on a conventional ventilator. A second dose of surfactant, i.e., Neosurf, was administered. After 24 hours, the respiratory distress settled, and the baby was weaned off to CPAP by 36 hours of life.

After 12 hours of extubation, the respiratory distress increased mandating re-intubation. Septic screen came out negative. There was persistent desaturation with blood gas suggestive of respiratory acidosis for which the conventional ventilation setting was increased gradually to a maximum of Fio2 of 100%, PEEP of 6, and peak inspiratory pressure (PIP) of 24. The baby was shifted to high-frequency ventilation thereafter. The baby failed to show improvement despite adequate ventilation and succumbed due to refractory respiratory failure.

**Figure 2:**
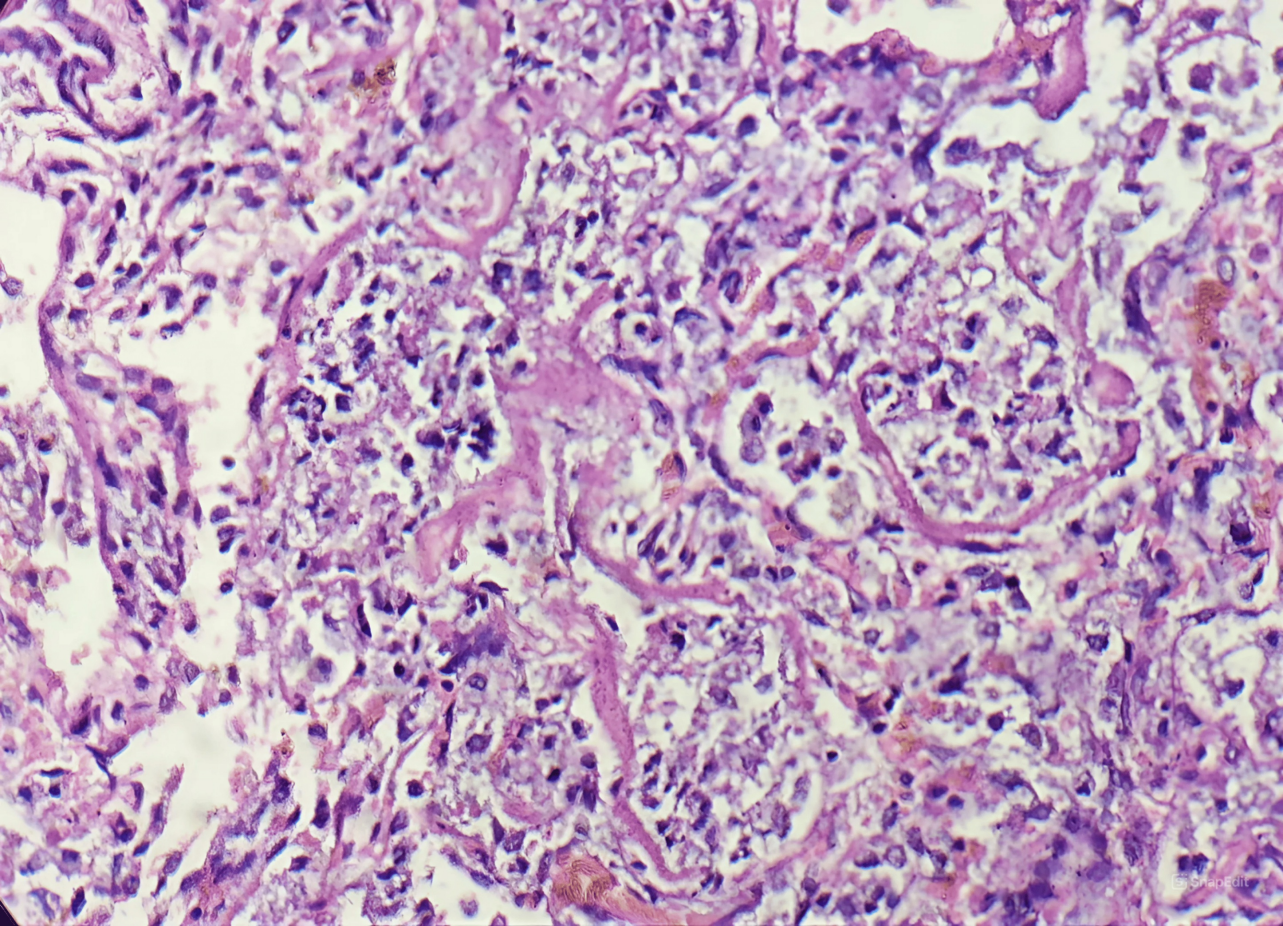
Lung biopsy showing the alveoli filled with hyaline matter and preserved lung architecture characteristic of pulmonary alveolar proteinosis

Postmortem lung biopsy was done suspecting surfactant deficiency due to previous neonatal deaths and nonresponse to conventional treatment with maximum ventilatory support. The biopsy was suggestive of diffuse alveolar damage with PAS-positive hyaline proteinaceous material lining the alveoli and preserved lung architecture suggestive of congenital alveolar proteinosis as depicted in Figure 2.

Due to financial constraints, the genetic mutation workup could not be carried out for this case. The parents were advised for genetic counselling before planning next pregnancy.

## Discussion

Congenital alveolar proteinosis is a rare genetic disorder with a reported prevalence of 0.1 per 100,000 with a characteristic deposition of abnormal surfactant protein within the alveoli [4]. Surfactant is synthesized by type II alveolar cells which act by reducing surface tension in the alveoli, thus preventing its collapsing during exhalation.

The exact cause of congenital PAP remains unclear although the widely accepted etiology is a mutation in surfactant protein B or C leading to intra-alveolar deposition of these abnormal nonfunctioning proteins as seen in our case on lung biopsy. Recently, a mutation in alpha or beta chains of GM-CSF has been identified in congenital PAP, although it is usually seen in adult forms of PAP [5].

A family history of similar respiratory issues in sibling or consanguineous marriage points toward an autosomal recessive transmission [4]. A history of consanguinity is an important clue which is usually present in 35% of the cases and was present in our case too [6].

According to available literature, there is a significant time gap between the onset of symptoms and the diagnosis of a patient of PAP which is around 4.5 months [6].

The symptoms are usually nonspecific presenting as growth retardation and progressive dyspnea in the majority of cases [7]. In our case, the presentation was very aggressive as symptoms were seen immediately after birth.

The diagnostic modalities available are X-rays, high-resolution computed tomography (CT) thorax, and bronchoalveolar lavage (BAL). CT thorax will show thickened septa with a characteristic crazy pavement pattern [8]. BAL will show a milky appearance of the washed fluid and a PAS-positive substance in the alveolar macrophage [3]. In our case, as the diagnosis was made on postmortem analysis, similar finding was seen in lung biopsy histology.

Treatment options are very limited and aimed at improving respiratory function and providing supportive care. A whole-lung lavage is the only intervention that is used widely and associated with long-term improvement [4]. The goal is to remove the proteinaceous material in alveoli and restore the gas exchange at the alveolar-capillary barrier, although this is of limited use in newborns because the use of large endotracheal tubes is associated with technical problems [9]. Other available less-used modalities are lung transplantation, gene therapy, GM-CSF administration.

Without lung transplantation, the mortality of congenital PAP is almost 100% [10]. Late-onset disease has better prognosis. Disease-specific five-year survival rates are 88% and 80% of deaths occur within the first year [7].

## Conclusions

PAP is a very rare disease causing respiratory distress; due to variations in the severity of the disease, and the pediatrician’s nonfamiliarity with PAP, its diagnosis is usually delayed or missed. It should be considered in cases with consanguinity, positive family history, and nonresponsiveness to standard treatment. Diagnosis can be confirmed on histopathologic analysis of samples from BAL or lung biopsy. Whole-lung lavage can be tried to improve survival and severe cases may need lung transplantation. The overall prognosis is poor and genetic counselling should be planned for subsequent pregnancies.
